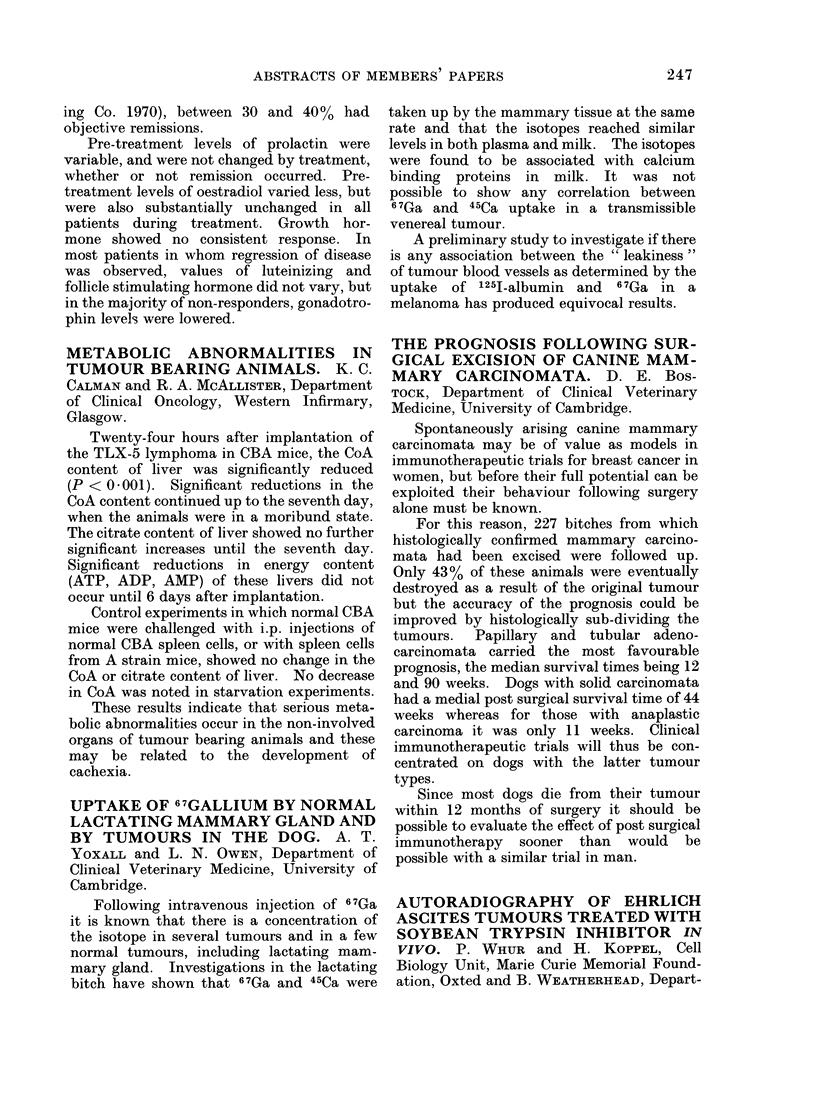# Proceedings: The prognosis following surgical excision of canine mammary carcinomata.

**DOI:** 10.1038/bjc.1975.179

**Published:** 1975-08

**Authors:** D. E. Bostock


**THE PROGNOSIS FOLLOWING SURGICAL EXCISION OF CANINE MAMMARY CARCINOMATA.** D. E. Bostock, Department of Clinical Veterinary Medicine, University of Cambridge.

Spontaneously arising canine mammary carcinomata may be of value as models in immunotherapeutic trials for breast cancer in women, but before their full potential can be exploited their behaviour following surgery alone must be known.

For this reason, 227 bitches from which histologically confirmed mammary carcinomata had been excised were followed up. Only 43% of these animals were eventually destroyed as a result of the original tumour but the accuracy of the prognosis could be improved by histologically sub-dividing the tumours. Papillary and tubular adenocarcinomata carried the most favourable prognosis, the median survival times being 12 and 90 weeks. Dogs with solid carcinomata had a medial post surgical survival time of 44 weeks whereas for those with anaplastic carcinoma it was only 11 weeks. Clinical immunotherapeutic trials will thus be concentrated on dogs with the latter tumour types.

Since most dogs die from their tumour within 12 months of surgery it should be possible to evaluate the effect of post surgical immunotherapy sooner than would be possible with a similar trial in man.